# Isogenic mice exhibit sexually-dimorphic DNA methylation patterns across multiple tissues

**DOI:** 10.1186/s12864-017-4350-x

**Published:** 2017-12-13

**Authors:** Helen McCormick, Paul E. Young, Suzy S. J. Hur, Keith Booher, Hunter Chung, Jennifer E. Cropley, Eleni Giannoulatou, Catherine M. Suter

**Affiliations:** 1 Victor Chang Cardiac Research Institute, 405 Liverpool Street, Darlinghurst, NSW 2010 Australia; 2 St Vincents Clinical School, Faculty of Medicine, University of New South Wales, Kensington, NSW 2052 Australia; 3 Zymo Research, Murphy Ave, Irvine, CA 92614 USA

**Keywords:** Epigenetics, DNA methylation, Sexual dimorphism, Gender, Tissue-specific methylation, RRBS

## Abstract

**Background:**

Cytosine methylation is a stable epigenetic modification of DNA that plays an important role in both normal physiology and disease. Most diseases exhibit some degree of sexual dimorphism, but the extent to which epigenetic states are influenced by sex is understudied and poorly understood. To address this deficit we studied DNA methylation patterns across multiple reduced representation bisulphite sequencing datasets (from liver, heart, brain, muscle and spleen) derived from isogenic male and female mice.

**Results:**

DNA methylation patterns varied significantly from tissue to tissue, as expected, but they also varied between the sexes, with thousands of sexually dimorphic loci identified. The loci affected were largely autonomous to each tissue, even within tissues derived from the same germ layer. At most loci, differences between genders were driven by females exhibiting hypermethylation relative to males; a proportion of these differences were independent of the presence of testosterone in males. Loci harbouring gender differences were clustered in ontologies related to tissue function.

**Conclusions:**

Our findings suggest that gender is underwritten in the epigenome in a tissue-specific and potentially sex hormone-independent manner. Gender-specific epigenetic states are likely to have important implications for understanding sexually dimorphic phenotypes in health and disease.

**Electronic supplementary material:**

The online version of this article (doi: 10.1186/s12864-017-4350-x) contains supplementary material, which is available to authorized users.

## Background

Sexual dimorphism, in which the two sexes exhibit different characteristics, affects a range of traits in animals. Almost all human diseases exhibit some component of sexual dimorphism, which can manifest as differences in disease incidence, age of onset, or severity of symptoms [1]. The discordance cannot be completely explained by genetic (i.e. sex chromosome) differences or the actions of sex hormones [2]. Despite increasing efforts in understanding the epigenetic contribution to complex disease [3], the contribution of epigenetic factors (such as DNA methylation) to sexual dimorphism is relatively underexplored.

There is evidence for gender differences in DNA methylation in various tissues in eutherian mammals. Unsurprisingly, the majority of differentially methylated loci occur on the X-chromosome, where they are hypermethylated in females relative to males [4]. However, sex-specific methylation also occurs on autosomes. A recent meta-analysis of Infinium 450 K array data from 76 individual studies identified gender-specific methylation at about 200 autosomal CpG sites in peripheral blood [4]. Gender-specific methylation differences have also been reported using 450 K array in human prefrontal cortex [5], saliva [6], and pancreatic islets [7], and by reduced representation bisulphite sequencing (RRBS) in mouse liver [8]. A notable recent study used RRBS to find gender-specific methylation at 160 autosomal loci in mouse liver [9]. In this study, a bias towards hypomethylation in males was dependent on testosterone exposure during puberty, which was coincident with loss of methylation. This study suggests that there is an interaction between sex hormones and epigenetic marks, but whether gender-specific marks might exist independent of hormonal or other postnatal factors is unknown.

Given that the bulk of de novo DNA methylation in mammals takes place during the very early stages of embryogenesis, it is possible that at least some gender methylation differences are specified early in development, or are even innate. Such gender differences would be consistent across different tissues, possibly even tissues derived from different germ layers. Here we have explored this possibility by performing RRBS on male and female mouse tissues derived from each of the three germ layers: liver (endoderm), heart (mesoderm), and brain (ectoderm). We find sexually dimorphic, differentially methylated loci in all tissues. By combining our analyses with publically available data, we find that in the liver, a proportion of loci exhibit gender-specific methylation in the absence of testosterone. Very few gender-specific differences are shared among tissues, even those from the same germ layer.

## Results

### Gender is predicted by DNA methylation patterns in the mouse liver

We generated snapshots of genome-wide cytosine methylation patterns with reduced representation bisulphite sequencing (RRBS). RRBS captures only ~1% of the mammalian genome, but the captured fraction is highly enriched for functional regions such as CpG islands, shores, and other gene regulatory elements. Here we used an enhanced RRBS protocol to produce methylomes from the livers of six female and six male isogenic C57BL/6 J mice. Overall, data quality was very high, as were inter-sample correlations (Additional file 1: Figure S1). For all analyses we considered only those CpGs that were present at >10× coverage in all 12 samples. To combine statistical evidence of neighbouring CpGs, we calculated DNA methylation levels in tiles of 100 bp across the genome (resulting in 820,388 tiles covered across all 12 samples).

**Fig. 1:**
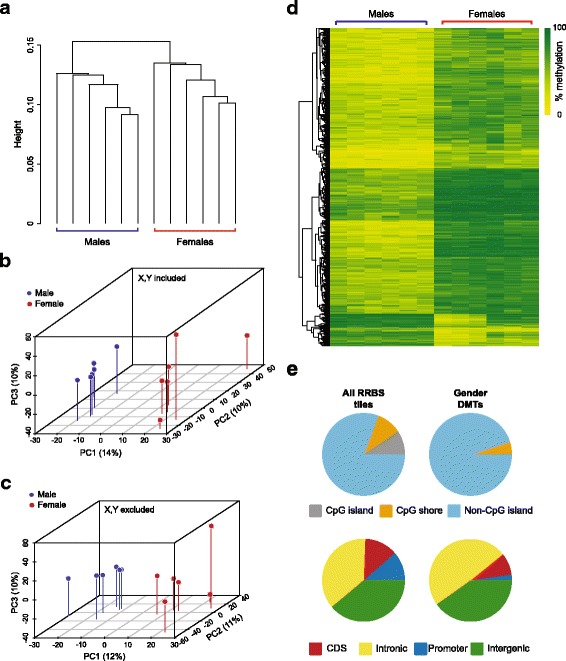
Autosomal DNA methylation patterns in the liver distinguish gender. **a** Dendrogram showing results of unsupervised hierarchical clustering of liver RRBS data from six males and six females with sex chromosomes excluded. **b**, **c** Pseudo-3D principal component analysis (PCA) plots of the first three principal components of liver RRBS data as in (a) with sex chromosome data included (b), or removed (c); males are shown in blue, females in red. **d** Clustered heat map of differentially methylated tiles (DMTs) identified in the liver of males versus females. **e** CpG island (top) and genomic annotation (bottom) of CpG tiles present in all liver RRBS data (left) and gender DMTs (right)

Unsupervised hierarchical clustering separated samples by gender, even when data from sex chromosomes was removed (Fig. 1a). Principal components analysis (PCA) also showed a distinct spatial clustering of samples by gender irrespective of whether sex chromosomes were included (Fig. 1b) or not (Fig. 1c). We identified regions of difference between genders using methylKit [10]. We found 1093 tiles that were differentially methylated (*q*-value < 0.01 and ≥ 25% methylation difference) between males and females (Fig. 1d; Additional file 2: Table S1). Despite the huge enrichment for CpG islands in our RRBS libraries, the majority of differentially methylated tiles (DMTs) were found outside of CpG islands, in both genic and intergenic regions (Fig. 1e). Around a third of the DMTs overlapped with ENCODE liver H3K4me1 and H3K27Ac peaks, a highly significant enrichment (both *p* < 1 × 10^−4^). This is consistent with many of the intergenic DMTs residing in active enhancers.

We chose a subset of ten DMTs to validate by COBRA [11]. All but one of the ten loci showed a difference in methylation levels between males and females as predicted by the RRBS signal (Additional file 3: Figure S2). This experimental validation indicates that our RRBS and informatics strategy detects gender-specific differences with high confidence.

### Gender DMTs in the liver can be testosterone independent

**Fig. 2:**
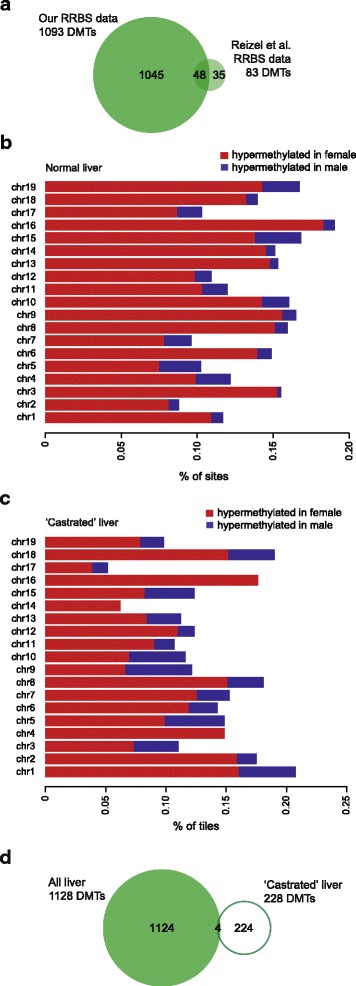
Gender-specific methylation in the liver does not require testosterone. **a** Venn diagram showing overlap of gender DMTs in the liver identified by this study and our reanalysis of Reizel et al. [9]. **b** Bar plot of gender DMTs from our liver RRBS data showing % of tiles hypermethylated in females (red) and males (blue) across all autosomes. **c** Bar plot of gender DMTs between normal females and castrated males showing % of tiles hypermethylated in females (red) and males (blue) across all autosomes. **d** Venn diagram showing overlap of all liver gender DMTs (both those identified here and those from Reizel et al. [9]) and testosterone independent DMTs (i.e. differentially methylated between normal females and castrated males)

We next reanalysed data from Reizel et al.*.* [9] (GEO Accession GSE60012), who previously identified 160 gender DMTs in the adult mouse liver. Using our bioinformatics pipeline with their liver dataset, we identified 83 autosomal gender DMTs, of which 48 (~58%) overlapped with the gender DMTs from our data (Fig. 2a, Additional file 2: Table S2). The difference in the number of DMTs identified can be at least partially attributed to the difference in dataset size (our liver dataset contained 820,388 tiles, and Reizel et al’s contained 167,462 tiles). Like Reizel and colleagues, we found that the majority of gender DMTs in the liver could be attributed to hypermethylation in females relative to males; this was true across all autosomes (Fig. 2b). Interestingly, the gender DMTs identified in the liver by Reizel et al. were absent from the liver of males who were castrated when young, and could be reconstituted in castrated males with exogenous testosterone administration [9]. This implies that gender DMTs in the liver are testosterone-dependent. We extended these observations by performing an unbiased comparison of the Reizel et al. females with the castrated males, using our own informatics pipeline. In doing so we were able to identify 228 gender DMTs, despite the absence of testosterone in the males (Additional file 2: Table S3). These testosterone-independent gender DMTs were also heavily skewed towards hypermethylation in females (Fig. 2c). Only four of these DMTs are also found in our set of 1093 DMTs (Fig. 2d), although almost all the tiles in the castrated male dataset were also represented in our dataset. This suggests that there are additional factors beyond testosterone that are able to specify gender-specific methylation patterns.

### Heart and brain also harbour gender DMTs

**Fig. 3:**
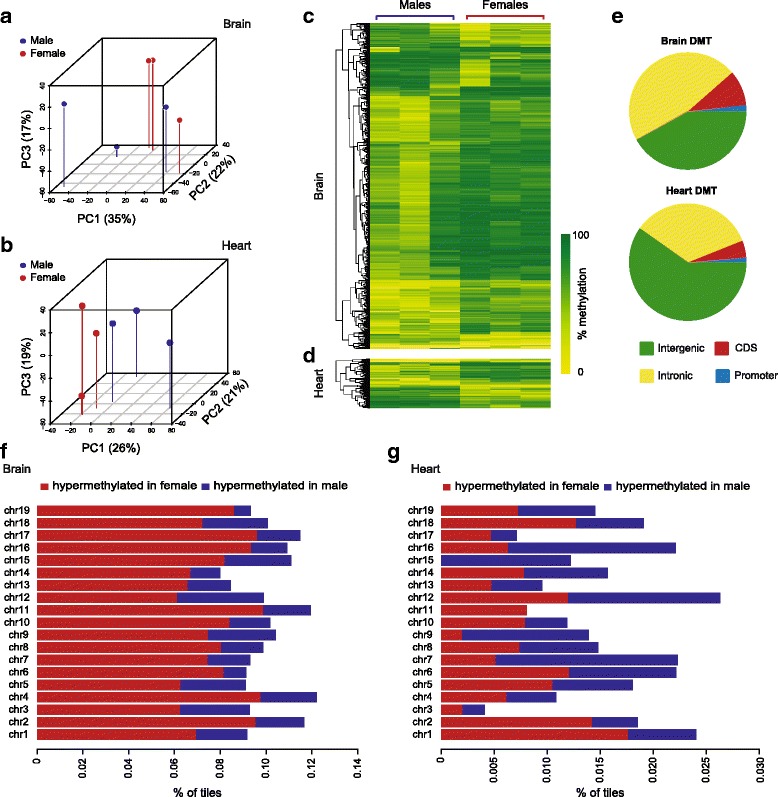
Mouse heart and brain also harbour gender-specific methylation. **a**, **b** Pseudo-3D PCA plots of the first three principal components of RRBS data from brain (a) and heart (b); males are shown in blue, females in red. **c**, **d** Heat maps of differentially methylated tiles (DMTs) identified in the brain (**c**) and heart (**d**) of males versus females. **e** Genomic annotations of brain and heart gender DMTs. **f**, **g** Bar plot of gender DMTs from brain (**f**) and heart (**g**) showing % of tiles hypermethylated in females (in red) and males (blue) across all autosomes

The robust gender differences observed in the mouse liver in this study and others, along with reports of gender bias in human non-liver tissues such as peripheral blood leukocytes, prompted us to ask whether the gender DMTs might be conserved across tissues. Given that some DMTs in liver appear to be sex hormone-independent, it is possible that these differences in methylation arise in the germline, or very early in development. To address this possibility we interrogated the methylomes of brain and heart from three of the males and three of the females used for liver analysis. We chose these tissues as they derive from different germ layer origins (heart, mesoderm; brain, ectoderm; liver, endoderm). Unlike the liver methylome, analysis of the brain methylome by hierarchical clustering or PCA did not separate the genders, even when sex chromosomes were included (Fig. 3a); likewise in the heart we observed no distinct clustering, although the genders separate very slightly on the first principal component (PC1; Fig. 3b). Despite the lack of unsupervised clustering by gender, both heart and brain harboured gender DMTs. Applying the same stringent parameters as used for the liver analysis, we identified 957 autosomal DMTs in the brain (Fig. 3c; Additional file 2: Table S4), and 145 in the heart (Fig. 3d; Additional file 2: Table S5). While the brain gender DMTs exhibited, like the liver, a clear bias towards hypermethylation in females (Fig. 3f), this bias was absent from the gender DMTs in the heart (Fig. 3g). The gender DMTs in both tissues were again mostly outside CpG islands, in intronic and intergenic regions (Fig. 3e). Like the liver DMTs, the brain DMTs were significantly associated with enhancer regions (*p* < 1 × 10^−4^), albeit with less fraction of overlap (~5% of DMTs); but the heart DMTs were not (*p* = 0.74).

### Gender DMTs are tissue specific

**Fig. 4:**
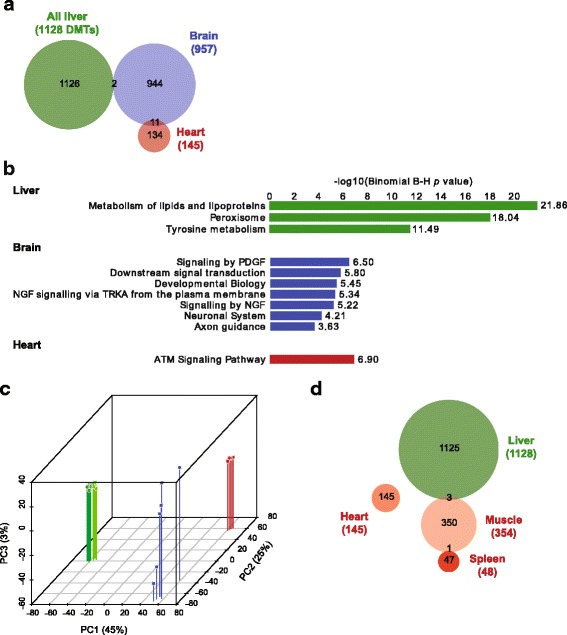
Gender DMTs are largely tissue autonomous. **a** Venn diagram showing overlap of gender DMTs from liver, brain and heart. **b** Molecular functions overrepresented by regions harbouring gender DMTs in liver, brain and heart. **c** Pseudo-3D PCA plot of RRBS data from liver (green), brain (blue) and heart (red). **d** Venn diagram showing overlap of gender DMTs from spleen, heart, skeletal muscle and liver

We then asked whether there was any overlap of the gender DMTs we identified among tissues of distinct germ layer origin that might suggest that they were inborn. Despite the large number of DMTs identified across the three tissues in total we found almost no overlap (Fig. 4a). Only two liver gender DMTs were common to the brain, and no liver DMT was common to the heart (although 11 of the 145 heart DMTs were also differentially methylated in the brain). We determined functional pathways associated with the DMTs in each tissue; in most cases, significantly enriched pathways were functionally related to the relevant tissue (Fig. 4b). This may not be surprising given that the DMTs tend to be over-represented in enhancer regions. PCA of all of our RRBS data across all tissues confirmed that methylation patterns overall are tightly associated with tissue type (Fig. 4c); it is interesting to note that methylation patterns in the brain are much more variable among isogenic individuals than the other tissues examined.

While this analysis suggests that gender DMTs do not arise in the germline, we considered whether gender DMTs might be common to tissues of the same germ layer origin (i.e. set early in development). We identified DMTs from additional mesodermal tissues studied by Reizel et al. (skeletal muscle and spleen from adult mice) to compare with our mesodermal (heart) tissue. We identified 354 and 48 gender DMTs in muscle and spleen respectively (Additional file 2: Tables S6 and S7), but these DMTs were exclusive to both each other and to the heart (Fig. 4d); this is despite almost all tiles from the muscle and spleen datasets being represented in our heart dataset. Taken together, these comparisons indicate that differential cytosine methylation between genders is almost entirely tissue-specific.

## Discussion

Here we confirm the existence of sex-specific cytosine methylation in the mouse liver and find that tissues derived from all three embryonic germ layers also exhibit sexually dimorphic patterns of methylation that are essentially idiosyncratic to a tissue. In the majority of instances, sex differences were manifest as a strong female bias towards hypermethylation, and this was the case in the liver even when the demethylating action of testosterone in males was removed. Our findings suggest that the DNA methylome undergoes gender differentiation in multiple tissues at some point after lineage specification, in response to tissue-dependent mechanisms.

Tissues of different origins displayed varying extents of gender-specific methylation: the brain harboured more than a thousand differentially methylated regions whereas the heart had only a few hundred. Sex-specific methylation in the brain was remarkable also because it occurred on a very high background of inter-individual variation. Extensive inter-individual variation in the methylome of the human brain has been recognised only recently, and attributed largely to a secondary influence of genetic variation [12]. However in our study all samples were derived from isogenic mice, indicating that at least some proportion of inter-individual variation in methylation patterns in the brain is purely epigenetic in nature, and sex-specific epigenetic signatures are overlaid on this. As our criteria for calling sex-specific methylation was strict (*q* < 0.01, methylation difference ≥ 25%), it is probable that thousands more sex-specific differences exist in the brain, albeit smaller in magnitude or restricted to specific cell subtypes.

Despite identifying many thousands of gender differences in cytosine methylation overall, we found very few that were in common among tissues. Even tissues originating from the same germ layer displayed a completely autonomous set of gender methylation differences. Taken together with the ontological analysis showing enrichment for tissue-specific pathways, this strongly suggests that gender-specific epigenetic differences are likely to have a tissue-specific function. This idea is supported by our finding that gender-specific methylation differences overlap significantly with histone markers of enhancer regions. It is also consistent with the recent finding that gender-specific methylation in the liver correlates with hepatic gene expression, particularly when the methylation differences occur in regions corresponding to tissue-specific enhancers [9]. The expression of more than a thousand genes in the mammalian liver is sex-dependent, which is not surprising given that sexual dimorphism in both steroid and drug metabolism underpin normal liver physiology [13]. Whether gender biases in methylation in heart and brain also influence their respective transcriptomes is not known, however the tissue-specific functional pathways we find suggest that this may well be the case.

The mechanisms underpinning gender-specific methylation are generally unexplored. However two recent studies have implicated testosterone in the process. In the mouse forebrain, male-specific methylation patterns can be induced in females by neonatal administration of exogenous testosterone [14]. In the liver, testosterone can trigger male-specific demethylation of certain regions; like females, castrated males maintain these regions as methylated [9]. However, not all gender-specific methylation can be attributed to the actions of testosterone: in an unbiased comparison we found more than 300 gender differences between the livers of females and castrated males. While these testosterone-independent gender differences account for a minority of all liver differences, they show the same strong tendency for hypermethylation in females, suggesting that other sex-specific *trans*-acting factors are also capable of effecting methylation dimorphism. Such factors may involve early and indirect actions of the sex chromosomes, as has been shown for early embryonic gene expression [15]. Female hormones such as estradiol are also capable of skewing gene expression patterns [16], and so may also be a contributor.

While the weight of our evidence supports widespread tissue-specific methylation differences between genders, our study is not without limitations and there are many unknowns that warrant further investigation. Comparative analysis of many tissues across multiple developmental stages (including embryonic, fetal, neonatal, and adult) would be required to establish the precise timing of establishment of gender differences, and the relationship to the changing hormonal milieu. Furthermore, RRBS allows the interrogation of methylation levels at only a representative fraction of the genome, and while this proportion is enriched for functionally relevant regions, whole genome bisulphite sequencing would be required to capture the full extent of gender-specific DNA methylation. Such studies will only become more feasible with the increasing affordability of whole genome sequencing.

Tissue-specific gender-bias in DNA methylation represents yet another aspect of sexual dimorphism but its function is currently unclear. While likely to reflect normal tissue physiology, such gender bias in epigenetic states may also have broader implications. Intrinsic gender bias in epigenetic state holds the potential to influence disease susceptibility and disease course, and modulate the response to environmental stressors. Gender differences in environmental epigenetic programming are pervasive [17] and our findings here suggest that at least some of the gender bias in induced phenotypes derives from baseline gender differences in epigenetic state.

## Conclusion

Our study of the DNA methylomes of multiple tissues of male and female mice indicates that sex significantly influences DNA methylation patterns in a tissue-specific manner. These findings provide a platform to better understand the role of DNA methylation in health and disease and have important implications for the study of complex and programmed phenotypes. Our findings underscore the need to consider both genders in epigenome-wide association studies, and reinforce the requirements related to the choice of tissue to study.

## Methods

### Mice and tissues

All animals in this study were generated contemporaneously. Mice were handled in accordance with good practice as defined by the National Health and Medical Research Council (Australia) Statement on Animal Experimentation, and requirements of state government legislation. The study was approved by the Garvan/St Vincent’s Animal Ethics Committee (#13/35). Six male and six female C57BL/6 J littermate pairs were selected at weaning and maintained on NIH-31 chow until 24 weeks of age. Tissues (brain, heart, liver) were collected and snap frozen prior to storage at −80°C until DNA extraction. DNA was extracted from a 5 mm coronal section of the rostral end of the brain, a 5 mm apical section of the heart, and the extreme caudal section of the left lobe of the liver.

### DNA extraction and RRBS

Frozen tissue sections were homogenised in lysis buffer (50 mM Tris-HCl pH 8.0, 100 mM EDTA, 1% SDS, 100 mM NaCl) and incubated with 400 μg/ml Proteinase K (Roche) overnight at 55°C, followed by phenol:chloroform extraction and ethanol precipitation. Genomic DNA was used for RRBS library preparation and sequencing through the Methyl-MiniSeq service of Zymo Research (Irvine, CA, USA).

### Differential methylation analysis of RRBS data

RRBS data from liver heart and brain generated for this study have been deposited in the Gene Expression Omnibus (GEO) under accession number GSE84573. eRRBS and mapping was performed by Zymo Research using proprietary methods. Bed data supplied was reformatted, and identification of differentially methylated cytosines and annotation was carried out in R with the methylKit package, v0.99.2 [10]. To combine statistical evidence of neighbouring CpGs, we calculated DNA methylation levels in tiles of 100 bp across the genome. For all analyses we considered only tiles that were present at > 10× coverage in all replicate samples and < 99.8th percentile of coverage values across all samples in the comparison. Hierarchical clustering was performed using correlation and the Ward’s minimum variance method. Identification of differentially methylated tiles was carried out with methylKit; thresholds for calling a difference were set at a methylation difference of ≥ 25%, and *q* ≤ 0.01. Genomic annotations of differentially methylated tiles were performed using the mm10 refgene table from UCSC Table Browser. Gene ontology analysis was performed with GREAT [18] using all default parameters with the exception that distal gene regulatory domains were set at a maximum of 100 kb. Histone modification enrichment analysis was performed by permutation of overlaps with replicated H3K4me1 and H3K27Ac peak files from ENCODE (liver, ENCSR000CDH and ENCSR000CAO; brain, ENCSR000CDF and ENCSR000CAE; heart, ENCSR000CDD and ENCSR000CAI); the overlap of DMTs was compared with the overlap observed using the same number of tiles randomly chosen from the dataset, over 10,000 iterations.

### Differential methylation analysis of RRBS data obtained from Reizel et al.

Datasets from liver, castrated liver, muscle and spleen from adult mice (20 weeks) were obtained from GEO Accession GSE60012. Sequencing reads were aligned to the mm10 genome with Bismark [19] and methylation percentage calls for each CpG site calculated using MethylKit. The sex chromosomes were removed. Our quality control based on pairwise correlations of all samples identified five liver outliers (three female and two male samples) that were excluded from further analysis. Similarly to the analysis of our RBBS data, to combine statistical evidence of neighbouring CpGs, we calculated DNA methylation levels in tiles of 100 bp across the genome, and considered only tiles that were present at > 10× coverage in replicate samples and < 99.8th percentile of coverage values. Given the different number of replicate samples used by Reizel et al. compared to our experimental design, we set this coverage restriction in at least across six samples in each group for liver female vs. male samples comparison, five samples in each group for females vs. castrated liver samples, six samples in each group for muscle female vs. male samples and three samples in each group for the spleen female vs. male samples comparison. Identification of differentially methylated tiles and annotations was carried out with methylKit; thresholds were set at a methylation difference of ≥ 25%, and *q* ≤ 0.01.

### Combined bisulphite restriction analysis (COBRA) validations

Ten loci identified as differentially methylated by RRBS were randomly chosen for independent verification by COBRA, with the additional criterion that they were in the vicinity of coding genes, long non-coding RNA or retrotransposon repeats. Primers were designed using MethPrimer [20] and sequences are available on request. Following bisulphite PCR and digestion with the relevant restriction enzyme, amplicons representing the fraction of methylated and unmethylated template were quantified using densitometry (Multi Gauge V2.3).

## Additional files

Additional file 1: Figure S1. Correlation matrices and corresponding correlation coefficients for each liver RRBS dataset. (PDF 251 kb)

Additional file 2: Table S1. DMTs between females and males in mouse liver. *P*-values and *q*-values were calculated using MethylKit. (XLSX 217 kb)

Additional file 3: Figure S2. COBRA vadlidation of candidate liver gender-specific DMRs. Bs-seq plots (top panels) show average percentage methylation values across each differentially methylated locus as assessed by RRBS for females (red) and males (blue). The CpG/s interrogated by COBRA are indicated by arrows. Graphs (bottom panels) show average percentage methylation in females (red) and males (blue) as assessed by densitometry of COBRA bands. Error bars represent SEM; *** *p* < 0.0001; NS, non significant. (PDF 634 kb)
