# Supplementary material for: Isogenic mice exhibit sexually-dimorphic DNA methylation patterns across multiple tissues
Source: BMC Genomics. 2017 Dec 13;18:966. doi: 10.1186/s12864-017-4350-x (PMC5729250; doi:10.1186/s12864-017-4350-x)
Supplement: Supplementary file 1 — Correlation matrices and corresponding correlation coefficients for each liver RRBS dataset. (PDF 251 kb) [file 12864_2017_4350_MOESM1_ESM.pdf]

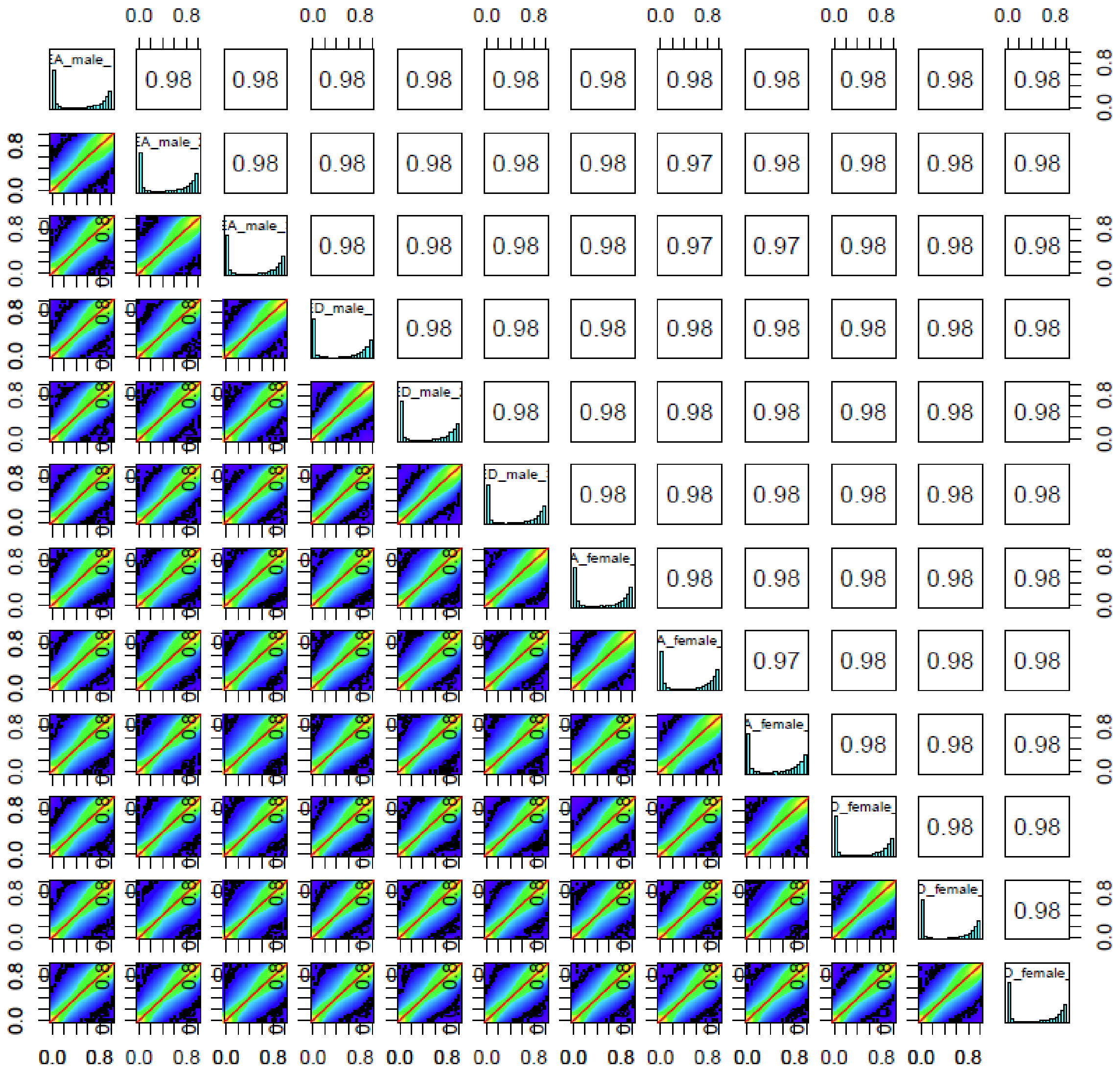

Additional File 1

Figure S1: Correlation matrices and corresponding correlation coefficients for each liver RRBS dataset.
