# Supplementary material for: Isogenic mice exhibit sexually-dimorphic DNA methylation patterns across multiple tissues
Source: BMC Genomics. 2017 Dec 13;18:966. doi: 10.1186/s12864-017-4350-x (PMC5729250; doi:10.1186/s12864-017-4350-x)
Supplement: Supplementary file 3 — COBRA vadlidation of candidate liver gender-specific DMRs. Bs-seq plots (top panels) show average percentage methylation values across each differentially methylated locus as assessed by RRBS for females (red) and males (blue). The CpG/s interrogated by COBRA are indicated by arrows. Graphs (bottom panels) show average percentage methylation in females (red) and males (blue) as assessed by densitometry of COBRA bands. Error bars represent SEM; *** p < 0.0001; NS, non significant. (PDF 634 kb) [file 12864_2017_4350_MOESM3_ESM.pdf]

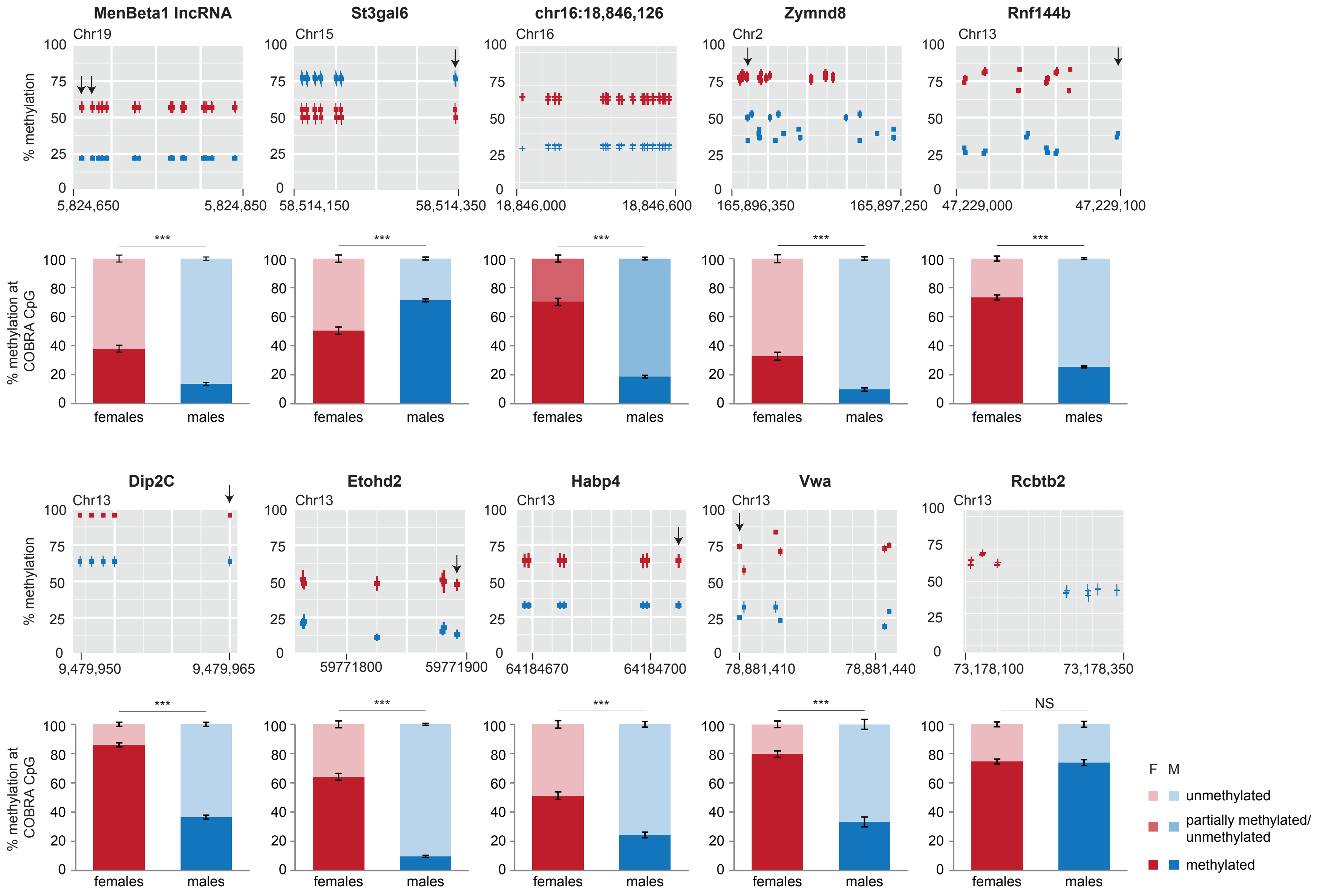

Figure S2: COBRA vadlidation of candidate liver gender-specific DMRs. Bs-seq plots (top panels) show average percentage methylation values across each differentially methylated locus as assessed by RRBS for females (red) and males (blue). The CpG/s interrogated by COBRA are indicated by arrows. Graphs (bottom panels) show average percentage methylation in females (red) and males (blue) as assessed by densitometry of COBRA bands. Error bars represent SEM; *** $p < 0.0001$; NS, non significant.
